# Combined Genome-Wide Association Study and Linkage Analysis for Mining Candidate Genes for the Kernel Row Number in Maize (*Zea mays* L.)

**DOI:** 10.3390/plants13233308

**Published:** 2024-11-26

**Authors:** Jiao Kong, Fuyan Jiang, Ranjan K. Shaw, Yaqi Bi, Xingfu Yin, Yanhui Pan, Xiaodong Gong, Haiyang Zong, Babar Ijaz, Xingming Fan

**Affiliations:** 1 College of Agronomy and Biotechnology, Yunnan Agricultural University, Kunming 650201, China; kongjiao2022@163.com; 2 Institute of Food Crops, Yunnan Academy of Agricultural Sciences, Kunming 650205, China; jiangfuyansxx@126.com (F.J.); ranjanshaw@gmail.com (R.K.S.); biyq122627@163.com (Y.B.); xingfuyin626@163.com (X.Y.); babarijazpbg@gmail.com (B.I.); 3 Institute of Resource Plants, Yunnan University, Kunming 650500, China; yahupan9988@163.com (Y.P.); xiaodonggong1998@163.com (X.G.); zhy15361180431@163.com (H.Z.)

**Keywords:** maize, kernel row number, GWAS, QTL mapping, candidate genes

## Abstract

Kernel row number (KRN) is one of the key traits that significantly affect maize yield and productivity. Therefore, investigating the candidate genes and their functions in regulating KRN provides a theoretical basis and practical direction for genetic improvement in maize breeding, which is vital for increasing maize yield and understanding domestication. In this study, three recombinant inbred line (RIL) populations were developed using the parental lines AN20, YML1218, CM395, and Ye107, resulting in a multiparent population comprising a total of 490 F9 RILs. Phenotypic evaluation of the RILs for KRN was performed in three distinct environments. The heritability estimates of the RILs ranged from 81.40% to 84.16%. Genotyping-by-sequencing (GBS) of RILs identified 569,529 high-quality single nucleotide polymorphisms (SNPs). Combined genome-wide association study (GWAS) and linkage analyses revealed 120 SNPs and 22 quantitative trait loci (QTLs) which were significantly associated with KRN in maize. Furthermore, two novel candidate genes, *Zm00001d042733* and *Zm00001d042735*, regulating KRN in maize were identified, which were located in close proximity to the significant SNP3-178,487,003 and overlapping the interval of QTL *qKRN3-1*. *Zm00001d042733* encodes ubiquitin carboxyl-terminal hydrolase and *Zm00001d042735* encodes the Arabidopsis Tóxicos en Levadura family of proteins. This study identified novel candidate loci and established a theoretical foundation for further functional validation of candidate genes. These findings deepen our comprehension of the genetic mechanisms that underpin KRN and offer potential applications of KRN-related strategies in developing maize varieties with higher yield.

## 1. Introduction

Maize (*Zea mays* L.) is cultivated globally and is one of the most significant food and feed crops worldwide. The primary objective for ensuring food security is to improve maize yield [1,2,3]. The kernel row number (KRN) is a vital determinant of maize yield, exerting a significant impact on yield and quality and serving as a key target for improvement of inbred lines [4,5,6]. KRN, a domesticated trait, exhibits substantial variation from its wild ancestors to cultivated maize [7] and is less influenced by environmental factors due to its high broad-sense heritability [8]. Therefore, studies have suggested that selection for KRN should be undertaken once advanced generation inbred lines demonstrate stable phenotypic performance [9,10]. KRN is a quantitative trait influenced by multiple genes and is controlled by additive and dominant genetic effects with over 100 quantitative trait locis (QTLs) identified for KRN [11,12]. A comprehensive understanding of the genetic and molecular basis of KRN is crucial for advancing maize yield, facilitating domestication, and promoting inflorescence development.

The genetic basis of complex quantitative traits is deciphered using molecular markers to map QTLs with the trait of interest. With the rapid development of molecular marker technologies, the integration of genome-wide association studies (GWAS) and linkage analysis for genetic mapping has become prevalent in molecular breeding research [13,14,15,16,17]. This approach harnesses the strengths of both methods [18,19], thereby facilitating the genetic enhancement of complex quantitative traits [20]. It has facilitated the identification of genetic loci about the various traits, such as ear weight and length [21,22], flowering time [23], and disease resistance [24] in maize.

Studies utilizing GWAS and linkage analysis have made significant progress in identifying candidate genes associated with KRN. Yang et al. [25] used composite interval mapping (CIM) to identify seven QTLs associated with KRN in an F_2_ population derived from B73 and SIYB1212 across two environments, which accounted for 6.78% to 36.76% of the phenotypic variance (PVE). Liu et al. [4] identified 31 single nucleotide polymorphisms (SNPs) associated with KRN, corresponding to 17 genomic loci by GWAS. Linkage analysis identified 33 QTLs linked to KRN in three F_2:3_ populations, including 21 QTLs common to multiple populations and environments. Chen et al. [26] constructed a set of four-way cross-mapping populations using four inbred maize lines with different grain types, and identified seven QTLs associated with KRN, explaining 4.47% to 11.24% of the phenotypic variance. Fei et al. [27] assessed KRN using 1617 RILs in four environments and identified five KRN-related QTLs, *qKRN1.1*, *qKRN2.1*, *qKRN4.1*, and new KRN-linked QTLs, *qKRN4.2* and *qKRN9.1*. Wang et al. [28] constructed four F_7_ RIL populations and through GWAS and linkage analyses, and, tiling GO analysis and alignment of different reference genomes, identified five SNPs associated with KRN and reported six novel candidate genes that regulate KRN. Despite the identification of some candidate genes for KRN, fewer genes have been successfully cloned. Liu et al. [29] conducted fine-mapping of a significant KRN-linked QTL, *KRN4*, which enhances maize yield by increasing KRN per ear. Han et al. [30] fine-mapped the major QTL *qKRN8* on chromosome 8 in a region of about 520 kb. An et al. [31] identified *Zm00001d016075* by fine-mapping *qKRN5.04* and validated its negative regulation of KRN by CRISPR-Cas9.

The advancement of tropical and subtropical germplasm resources is essential for developing core germplasms and addressing the breeding challenges in maize [32]. Tropical and subtropical maize germplasms display substantial genetic diversity, which remains inadequately explored and utilized, thus offering significant potential for identifying important candidate genes related to KRN. However, despite these advancements, there are still knowledge gaps and unanswered questions in KRN research. The precise genetic mechanisms underlying KRN in maize remain elusive, and the functional roles of candidate genes identified through GWAS and linkage analysis using tropical maize germplasm need further validation. Additionally, most previous studies have focused on temperate germplasm and individual genetic approaches, either GWAS or linkage analysis, without integrating the strengths of both methods to maximize the discovery of candidate genes. In this study, three maize inbred lines, AN20, YML1218, and CML395, demonstrating wide genetic variations in KRN were utilized as female parents, with AN20 and CML395 representing subtropical and tropical germplasms, respectively. These lines were crossed with the maize inbred line Ye107 to develop a multiparent population (MPP) consisting of three RIL (F9) subpopulations. Phenotypic data for KRN were collected from three distinct environments, and genome-wide SNPs were used to conduct GWAS and QTL mapping. The present study aimed to (1) identify SNPs and QTLs associated with maize KRN through combined GWAS and linkage analyses in an MPP population, and (2) identify candidate genes associated with KRN.

## 2. Results

### 2.1. Phenotypic Analysis for KRN

**Figure 1:**
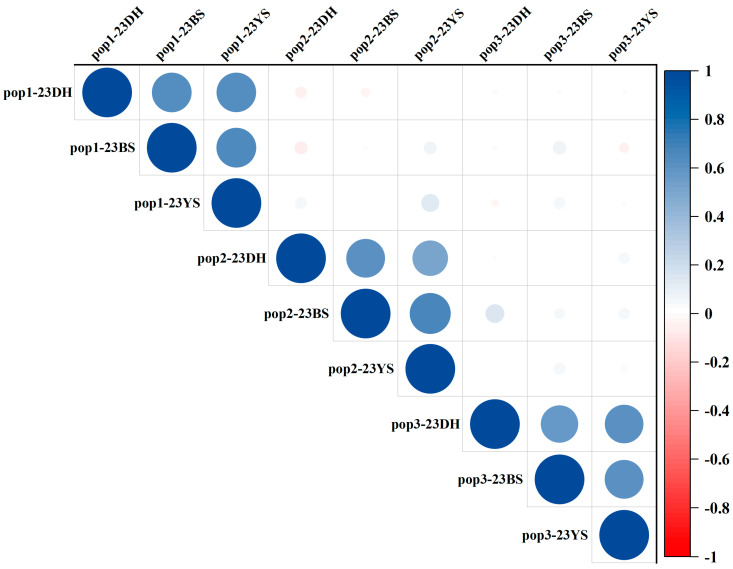
Correlation analysis diagram. Darker colors indicate stronger correlations between the environments of their respective subpopulations.

The KRN data of the three RILs subpopulations (pop1, pop2, and pop3) were collected across three locations, in Yunnan Province, China: Dehong Prefecture in 2023 (23DH), Baoshan City in 2023 (23BS), and Yanshan County in 2023 (23YS) and analyzed (Table 1). The analysis revealed that the mean KRN values for the three subpopulations ranged from 11.181 to 11.731, with coefficients of variation ranging from 12.33 to 16.96. Furthermore, the absolute values of skewness and kurtosis for three subpopulations were approximately 1, indicating that KRN across the populations followed a normal distribution, thus confirming that KRN is a quantitative trait. The broad-sense heritability for KRN in the three subpopulations ranged from 81.40% to 84.16%, and the correlation coefficients of RILs in different environments ranged from 0.512 to 0.680, suggesting that KRN is predominantly governed by genetic factors (Table 1, Figure 1). 

### 2.2. SNP Identification and LD Decay Analysis

**Figure 2:**
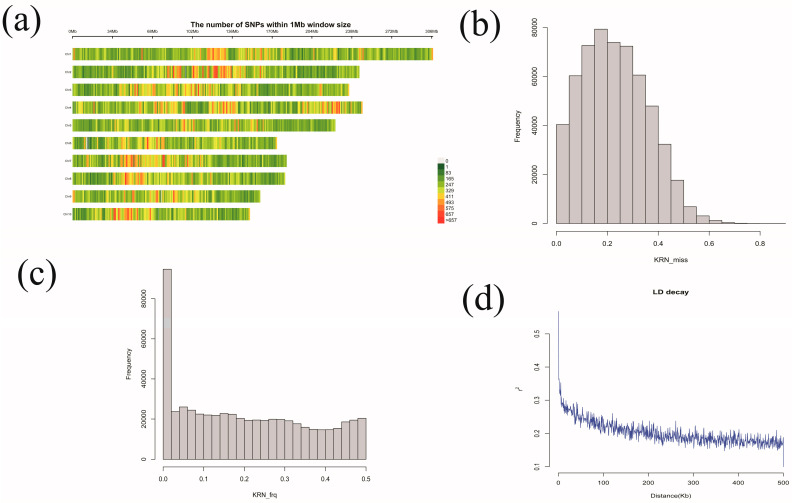
(**a**): SNP density distribution (SNP density in 1 Mb interval), (**b**): Distribution of the missing rate of the SNPs, (**c**): Distribution of MAF of SNPs, (**d**): LD decay plot of all the chromosomes.

A total of 569,529 high-quality SNPs were identified across the ten chromosomes of maize through genotyping-by-sequencing (GBS), as shown in the heat map (Figure 2a). The numbers of SNPs on chromosomes 1 to 10 were as follows: 455,704, 375,208, 355,799, 358,821, 348,005, 342,971, 341,859, 340,365, 335,177, and 103,809, respectively. Chromosome 1 had the highest numbers, while chromosome 10 had the lowest numbers. The SNPs with missing values of <0.2 and minor allele frequency (MAF) > 0.05 were selected for subsequent GWAS analysis. The distribution of SNPs for missing rate and MAF are presented in Figure 2b and Figure 2c, respectively.

The identified SNPs were used to assess the linkage disequilibrium (LD) decay, which was found to decrease rapidly as the physical distance between SNPs increased. The estimated LD decay was approximately 20 kb at a threshold (r^2^) of 0.3, so 20 kb upstream and downstream of the SNP was chosen as the criterion for screening candidate genes (Figure 2d).

### 2.3. Population Stratification Analysis

**Figure 3:**
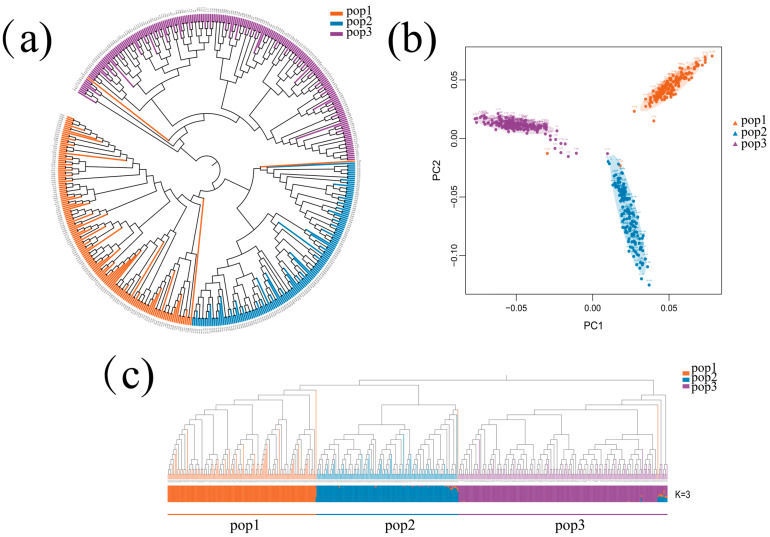
(**a**) Phylogenetic analysis; (**b**) PCA; (**c**) Bayesian clustering diagram of 409 RILs at K = 3. Different colors indicate different subpopulations: orange: pop1; blue: pop2; purple: pop3.

The phylogenetic tree clustered the 490 RILs into three groups, each colored differently (Figure 3a), and principal components analysis (PCA) results also show that the RILs were grouped into three major clusters (Figure 3b). Population structure analysis revealed that at K = 3, the RILs were clustered into three groups, with 148 RILs in pop1 (AN20 × Ye107), 138 RILs in pop2 (YML1218 × Ye107), and 204 RILs in pop3 (CML395 × Ye107) (Figure 3c). Overall, the three results were consistent with the experimental design of this study.

### 2.4. Genome-Wide Association Analysis for KRN

**Figure 4:**
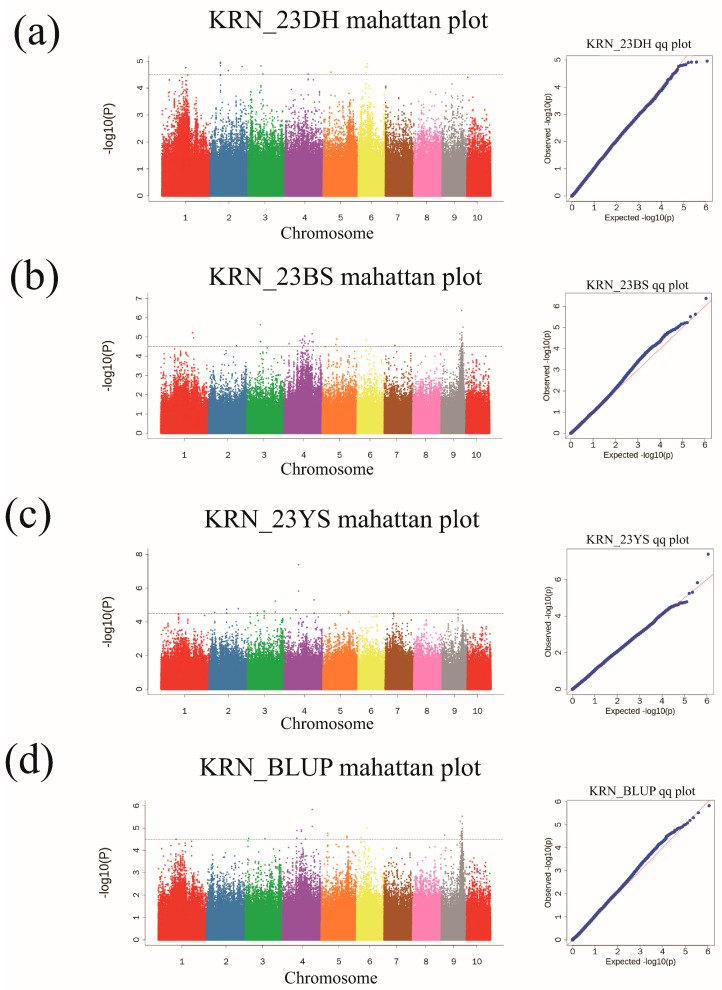
Manhattan map (**left**) and Q–Q plots (**right**) in the (**a**) 23DH environment, (**b**) 23BS environment, (**c**) 23YS environment, and (**d**) BLUP values.

**Figure 5:**
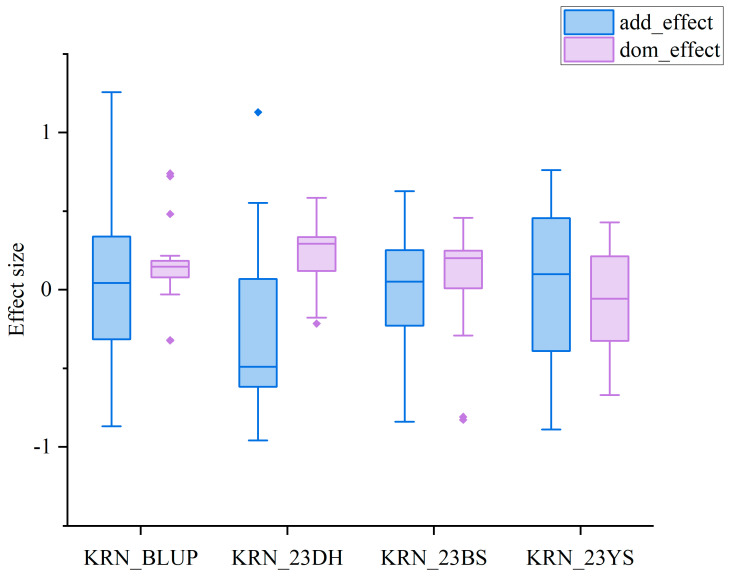
Box plots depicting the additive and dominant effects of significantly associated SNPs identified in different environments, blue indicates additive effect, purple indicates dominant effect.

GWAS was conducted using 569,529 high-quality SNPs combined with the mean KRN and the best linear unbiased prediction (BLUP) values of the 490 RILs of the MPP. The mixed linear model (MLM) model in GEMMA was used to identify the loci associated with KRN. The population structure and kinship matrix were incorporated as covariates to reduce false positives. Through GWAS, 120 SNPs significantly associated with KRN were identified (Figure 4, Table S1) across the environments. These SNPs exhibited both positive and negative additive and dominant effects, with a few exhibiting partially epistatic effects (Figure 5, Table S1). In the 23DH environment, 15 significant SNPs were identified on chromosomes 1, 2, 3, 4, 5, and 6, with the PVE ranging from 4.62% to 6.81% (Figure 4a, Table S1). In the 23BS environment, 45 significant SNPs were identified on chromosomes 1, 2, 3, 4, 5, 6, 7, and 9, with the PVE ranging from 3.82% to 10.06% (Figure 4b, Table S1). In the 23YS environment, 23 significant SNPs were identified on chromosomes 2, 3, 4, 5, 7, and 9, with the PVE ranging from 4.92% to 8.56% (Figure 4c, Table S1). For the BULP values, 37 significant SNPs were identified on chromosomes 3, 4, 5, 6, and 9 (Figure 4d, Table S1), with the PVE ranging from 3.53% to 10.61%.

### 2.5. Candidate Genes Identified in GWAS Analysis

Through GWAS analysis, 120 SNPs associated with KRN were identified, and 63 candidate genes potentially were identified on chromosomes 1, 2, 3, 4, 5, and 9 (Table S2). Of these, 44 candidate genes were functionally annotated, while the functions of the remaining 19 candidate genes remain unknown.

### 2.6. Linkage Map Construction and QTL Mapping for KRN

In this study, high-density linkage maps for the three populations (pop1, pop2, pop3) were constructed, using 847 bins in pop1, 829 bins in pop2, and 2021 bins in pop3. The total genetic distance of the genetic map of pop1 was 701.28 cM, with an average genetic distance of 0.83 cM. Chromosome 1 was the longest at 117.13 cM, while chromosome 2 was the shortest at 41.17 cM. The total genetic distance of the linkage map for pop2 was 1368.4 cM, with an average genetic distance of 1.65 cM. Chromosome 5 was the longest at 233.94 cM, while chromosome 9 was the shortest at 45.3 cM. The total genetic distance of the linkage map for pop3 was 4593.45 cM, with an average genetic distance of 2.27 cM. Chromosome 4 was the longest at 651.73 cM, and chromosome 9 was the shortest at 268.6 cM (Figure S1, Table S3).

During QTL mapping, 22 QTLs associated with KRN were identified on chromosomes 1, 2, 3, 5, 6, 7, 8, 9, and 10 (Figure S2, Table 2). In pop1, eight QTLs were identified in the 23DH, 23YS, and BLUP environments, with LOD ranging from 2.831 to 3.950. These QTLs explained 7.53–11.39% of the phenotypic variance (R^2^) and exhibited predominantly positive additive effects. In pop2, four QTLs were identified in the 23YS and BLUP environments, with LOD ranging from 2.803 to 3.999. These QTLs explained between 7.92% and 12% of the R^2^, with all the identified QTLs showing positive additive effects. In pop3, ten QTLs were identified in the 23DH, 23YS, 23YS, and BLUP environments, with LOD ranging from 2.567 to 6.367. These QTLs explained R^2^ between 5.45% and 15.96%. Among these, only *qKRN6-2*, *qKRN8-2*, and *qKRN7-4* showed positive additive effects, whereas remaining QTLs exhibited negative additive effects. 

### 2.7. Combined GWAS QTL Mapping and QTL Mapping to Identify Candidate Genes

**Figure 6:**
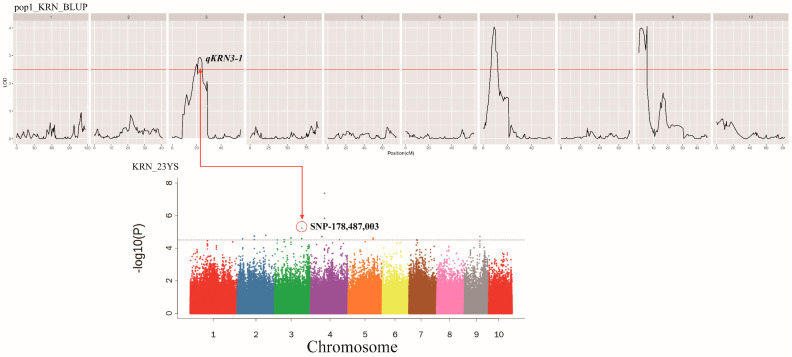
The SNP overlapping the QTL intervals identified through combined QTL mapping and GWAS, different colors indicate the location of SNPs on chromosomes 1–10.

Comparison of the results of GWAS and QTL mapping revealed that SNP3-178,487,003, located on chromosome 3 identified through GWAS in the 23YS environment, overlapped with the QTL interval of *qKRN3-1* mapped in pop1 for the BLUP values (Figure 6). This SNP explained 7.23% of the phenotypic variance, exhibiting both positive additive and dominant effects. Based on the co-localization analysis, *Zm00001d042733* and *Zm00001d042735* (Table 3) were identified. *Zm00001d042733* was located 313 bp downstream of SNP3-178,487,003 and *Zm00001d042735* was located at 6940 bp downstream of SNP3-178,487,003. Functional annotation revealed that *Zm00001d042733* is related to ubiquitin carboxyl-terminal hydrolase (UCH), while *Zm00001d042735* encodes the Arabidopsis Tóxicos en Levadura (ATL) family of proteins.

### 2.8. Haplotype Analysis

**Figure 7:**
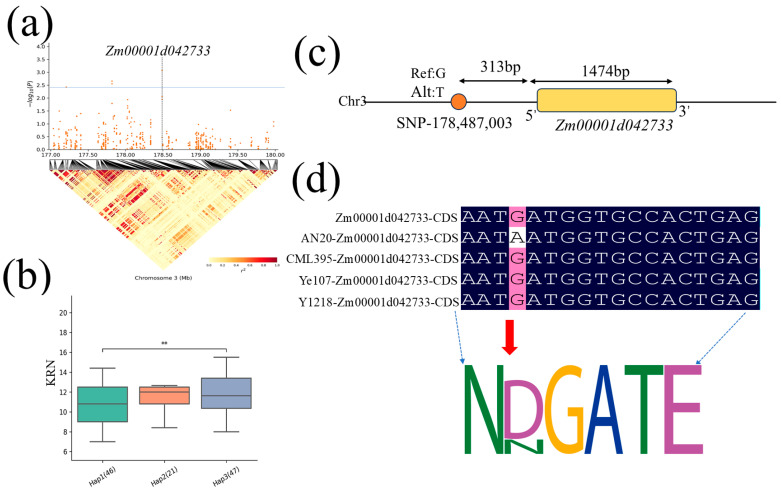
Haplotype location and sequence comparison analysis. (**a**) Positions of the candidate gene *Zm00001d042733* on chromosome 3. (**b**) The haplotype distribution of *Zm00001d042733* in the MPP, ** denotes *p* < 0.001. (**c**) The relative position of *Zm00001d042733* and the associated SNP. (**d**) The single nucleotide variation among the parental lines AN20, CML295, YML1218, and Ye107 in the CDS region causing change in amino acid (from aspartic acid to asparagine) and protein motif.

**Figure 8:**
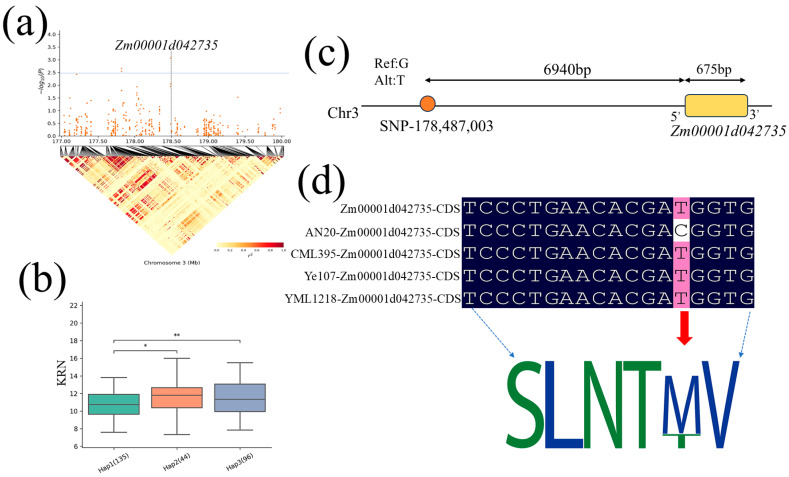
Haplotype location and sequence comparison analysis. (**a**) Positions of the candidate gene *Zm00001d042735* on chromosome 3. (**b**) The haplotype distribution of *Zm00001d042735* in the MPP, * denotes *p* < 0.05, ** denotes *p* < 0.001. (**c**) The relative position of *Zm00001d042735* and the associated SNP. (**d**) The single nucleotide variation among the parental lines AN20, CML295, YML1218, and Ye107 in the CDS region causing change in amino acid (from methionine to threonine) and protein motif.

Haplotype analysis of *Zm00001d042733* and *Zm00001d042735* identified through co-localization analysis was conducted. The gene *Zm00001d042733* has three major haplotypes (Figure 7a, Table 4). The frequency distribution of each haplotype in the MPP was as follows: Hap1 (46), Hap2 (21), and Hap3 (47) (Figure 7b). Among these three haplotypes, there were significant differences for KRN between Hap1 (CGTT) and Hap3 (CACG), with Hap3 (CACG) considered to be the superior haplotype of *Zm00001d042733* to increase KRN in maize. This gene is located 313 bp downstream of SNP3-178,487,003 (Figure 7c). Sequence comparison of this gene among the parental lines revealed a G/A mutation in AN20, resulting in an amino acid substitution (from aspartic acid to asparagine), which alters the protein motif (Figure 7d). The other candidate gene, *Zm00001d042735*, also has three major haplotypes (Figure 8a, Table 4). The frequency distribution of each haplotype was as follows: Hap1 (135), Hap2 (44), and Hap3 (96) (Figure 8b, Table 5). Of these three haplotypes, Hap1 (CTCG) and Hap2 (CCCG) differed, and Hap1 (CTCG) and Hap3 (TTCG) differed significantly, with Hap1 showing the lowest KRN. Thus, Hap3 (CACG) was considered to be the superior haplotype of *Zm00001d042735* to increase KRN in maize (Figure 8b). This gene is located 6940 bp downstream of SNP3-178,487,003 (Figure 8c). Comparison of the sequences of this gene among the parental lines revealed a specific T/C mutation in AN20, resulting in an amino acid substitution (from methionine to threonine), which alters the protein motif (Figure 8d). 

## 3. Discussion

### 3.1. The Feasibility of Combined GWAS and QTL Mapping

QTLs with smaller effects are challenging to identify during linkage analysis due to the low resolution in the classical bi-parental populations. Additionally, the limited genetic diversity in segregating populations often hinders the identification of rare alleles [33,34]. Similarly, population structure and low-frequency alleles limit the ability of GWAS to detect phenotypic effects of rare alleles [35], often resulting in higher false positives [36], which complicates the genetic study of complex traits in plants. In this context, combining GWAS and QTL mapping can compensate for the limitations of these mapping approaches, thereby enhancing QTL detection. This combined approach effectively overcomes the inherent constraints of linkage, and LD mapping enables the precise identification of rare or small-effect QTLs and helps in controlling false positives [5,37]. This approach is widely used to identify disease-resistant genes and elucidate the genetic basis of the traits of interest [19,38,39,40]. This approach has proven effective in dissecting the genetic architecture of quantitative traits in plants [41,42,43]. Previous studies have successfully identified several functional loci associated with KRN using this method [26,27,28]. In this study, 569,529 SNPs were used to identify potential functional loci and candidate genes regulating KRN using a combined GWAS and QTL mapping approach. Our results indicate that KRN is controlled by multiple loci with minor effects. Consequently, the loci detected by this approach were used to identify candidate genes for further functional studies, which will be applicable to further studies of KRN-related genes. 

### 3.2. The Comparison of This Study with Previous Studies

Previous studies have emphasized that additive and partial dominant effects are crucial to regulate grain development [44]. In our study, the significant SNPs exhibited additive, dominant, and epistatic effects. Our findings revealed SNPs with additive and partially dominant effects; however, the epistatic effect was not significant, which was consistent with the findings of previous studies by Liu et al. and Xiao et al. [4,45]. These authors showed that loci exhibiting additive and partially dominant effects exert a substantial influence on the genetic architecture of KRN.

By combining GWAS and QTL mapping results, two candidate genes for KRN were identified (Table 4), both located on chromosome 3. Although these genes were located on the same chromosome as those in previous studies, their physical locations differed [46,47]. Several factors could be responsible for this. Firstly, variation in genetic background: The genetic background of the mapping population is an important factor contributing to inconsistency in QTL identification. Moreno-Gonzalez et al. observed significant differences in the estimation of QTL effects associated with markers in multiple regression analysis, particularly in hybrids and backcross lines developed by different strains [48]. Beavis et al. [49] demonstrated that differences in genetic background contributed to the variation in QTL detection between topcross and F_4_ populations. Li et al. [50] showed that two populations differed in the expression of certain QTLs by comparing the mapping results of the BC_2_F_2_ population and the F_2:3_ population. This suggests that genetic background influences QTL mapping and underscores the importance of utilizing diverse genetic backgrounds for QTL analysis. Secondly, the use of varieties and germplasms from different ecotypes: Tropical and subtropical maize germplasm, when compared with temperate germplasm, exhibited higher genetics, which provides rich genetic resources for the improvement of KRN. Prolonged adaptation to high temperature and humidity in tropical environments may result in variations in KRN in tropical and subtropical germplasms, affecting the mapping of KRN. For instance, Yuan et al. [51] revealed that tropical and subtropical maize germplasms influence key genetic variants and genes affecting maize yield and flowering time. In this study, two novel candidate genes regulating KRN were identified in the subtropical germplasm AN20, but not in the tropical germplasm CML395. This may be attributed to natural or artificial selection favoring the dominant alleles and their linked polymorphisms at higher frequencies, leading to a reduced genetic diversity and contributing to environmental adaptation and phenotypic changes. Thirdly, variation in the reference genomes: The use of different maize reference genomes, especially in heterozygous populations, may lead to differences in gene identification. The reliance on a single reference genome for GWAS can complicate the process of identifying and characterizing genes located within the presence–absence variation (PAV) loci [52]. Wang et al. [28] identified candidate genes associated with KRN that were mapped using different reference genomes by analyzing the relationship between genetic variation in KRN across different tropical maize parents. In this study, the B73 reference genome was used, and candidate genes identified differed from those in previous studies. This could be attributed to sequence variation between the tropical maize germplasm and B73, affecting the gene expression and functional studies. Therefore, these candidate genes are novel candidate genes which regulate KRN in maize.

### 3.3. The Functional Annotation of Candidate Genes Co-Located by GWAS and QTL Analysis

This study identified two candidate genes, *Zm00001d042733* and *Zm00001d042735*, located 313 and 6940 bp downstream of the SNP3-178,487,003, respectively. Among these, *Zm00001d042733* encodes a UCH enzyme, which belongs to a subclass of enzymes such as deubiquitinating enzymes (DUBs), and affects auxin-dependent developmental pathways in *Arabidopsis thaliana* through its deubiquitination activity [53]. Studies on the UCH subfamily have shown that Arabidopsis UCH1 and UCH2 influence the turnover of the Arabidopsis auxin resistance gene, which serves as a pivotal regulator within the auxin response pathway. This indicates that these enzymes directly regulate the deubiquitination of one or more ubiquitin substrates involved in auxin signaling [54]. Furthermore, studies have indicated that auxin signaling has a potential tissue-specific role in maize grain development [55,56,57]. Auxin promotes the formation of pistil primordium in the early developmental stage of floral organs, and participates in the maturation and morphological shaping of pistils in the later stage [58]. Research has shown that ETTIN is a pivotal transcription factor in auxin signaling pathway and regulates expression of genes related to auxin response [59]. Heisler et al. proposed that SPT promotes auxin signal transduction at the top of the pistil, and the *SPATULA* gene regulates pollen and stamen development [60]. 

*Zm00001d042735* encodes the ATL protein and belongs to the RING-H2 E3 subfamily, actively regulating a variety of growth and developmental processes [61,62]. Functional analyses of many ATLs have revealed their involvement in different pathways in plants, including defense responses, regulation of carbon and nitrogen balance during the transition to seedling stage after germination, and endosperm development [63]. Ding et al. [64] showed that 9 days after pollination, 12 *ZmATL* family genes including *ZmATL2* and *ZmATL23* were highly expressed in the tassel, and 33 genes including *ZmATL13* and *ZmATL1* were also significantly expressed. Functional analyses of many ATLs have revealed their involvement in different pathways in plants, including defense responses, regulation of carbon/nitrogen balance during the transition to seedling stage after germination, and endosperm development. *AtATL78* negatively regulates cold and drought stress responses. By regulating environmental stress responses, it enhances cold and drought tolerance, which in turn affects kernel formation and development [65]. Pagnussat et al. [66] showed that *AthATL49/MEE*, a Ds transposon insertion line, is essential for the development and function of Arabidopsis female gametophytes.

### 3.4. The Important Factors Affecting KRN 

As one of the key agronomic traits that determine the yield and adaptability of maize, KRN is affected by various factors, including environmental conditions and other ear-related traits. National and international studies have highlighted the significance of these factors in shaping and regulating KRN, offering important theoretical support for optimizing maize breeding and management [67,68]. Studies have shown that maize is susceptible to drought and heat stress to a greater extent than other cereals [69]. Senhom et al. [70] investigated the potential of maize hybrids under different moisture conditions during drought, and found that days to tasseling, days to silking, kernel number per row (KNPR), KRN, and hundred kernel weight (HKW) could serve as effective selection indexes for yield improvement in maize breeding under conditions of limited moisture. Furthermore, Duan et al. [71] observed significant differences in ear weight, KRN, ear diameter (ED), and HKW in different row spacing and plant density. The authors noted that the interaction among multiple panicle traits, with varying effects and sizes, jointly affected maize yield. Feng et al. [72] identified eight candidate genes related to the number of KRN, KNPR, and ED, which interacted to affect yield in maize. In summary, future research should integrate multiple factors to enhance the comprehensive understanding of the regulatory network controlling KRN. This would provide a scientific basis and technical support for precision breeding and adaptive improvement.

## 4. Conclusions

In this study, three RIL populations were developed using AN20, YML1218, CML394, and Ye107 to develop an MPP and study the genetic basis of KRN. Combined GWAS and bi-parental QTL mapping was employed to identify the co-localized loci and candidate genes regulating KRN. Using this method, two candidate genes, *Zm00001d042733* and *Zm00001d042735*, located 313 and 6940 bp downstream of the SNP3-178,487,003 were identified. *Zm00001d042733* encodes ubiquitin carboxy-terminal hydrolase, while *Zm00001d042735* encodes the Arabidopsis Tóxicos en Levadura protein family. These hydrolases and proteins are involved in the regulation of KRN through deubiquitination activity, signal transduction, and participation in cellular embryonic development. Further studies are needed to validate the functions of these genes using genome editing technologies, such as CRISPR/Cas9 to enhance the breeding efficiency. Additionally, combining transcriptomic, metabolomic, and epigenomic studies could provide deeper insights into the complex genetic mechanisms of KRN. Additionally, the novel findings of this study warrant further exploration to elucidate the genetic and molecular basis of KRN. This study has established a foundation for marker-assisted breeding of KRN and offers direction for the development of high-yielding maize varieties.

## 5. Materials and Methods

### 5.1. Experimental Materials and Population Development

**Figure 9:**
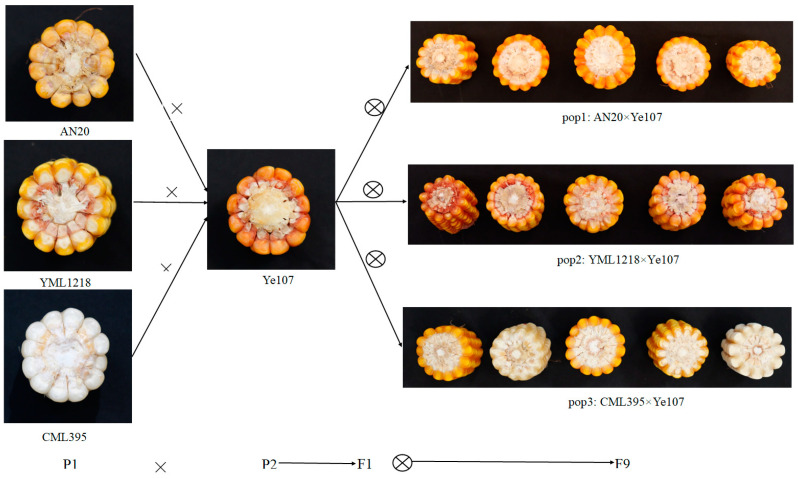
The scheme for developing RIL populations.

Three maize inbred lines AN20, YML1218, and CML395 with extensive genetic variation in KRN were used as the female parents, along with the elite temperate maize inbred line Ye107 as the co-parent. The pedigree, heterotic group, and ecological group of the four parents are shown in detail in Table 5. The F1s were generated by crossing these lines, which were successively self-crossed for eight generations by the single-seed descent method, resulting in an MPP consisting of three RIL populations: pop1, pop2, and pop3 (Figure 9). Among the 490 RILs, pop1 consisted of 148, pop2 comprised 138, and pop3 contained 204 RILs.

### 5.2. Field Experimental Design and Phenotyping

The field trials for KRN were conducted in three locations (23DH, 23BS, and 23YS) (Table 6). The experiment was conducted using a randomized block design with three replications in each location. The dimensions of each test plot were 4 m long, the distance between plants was 25 cm, and 14 plants were planted in each row. Standard fertilization and plant protection measures were carried out during the trial.

### 5.3. Phenotypic Data Analysis for KRN

Upon reaching maturity, the cobs were harvested and dried, and five cobs were randomly selected from each plot to measure the KRN of each RIL. The mean KRN of the 490 RILs of the MPP was subsequently calculated. The statistical analysis of KRN data was performed using the SPSS software (SPSS Statistics 26). Descriptive statistics, including the mean, min, max, SD, skewness, kurtosis, and CV were measured. The phenotypic data were analyzed graphically using the ORIGIN software (Origin 2022). The H^2^ was calculated as proposed by Knapp et al. [73].

$$H^{2}=\frac{{\sigma g}^{2}}{{\sigma g}^{2}+{\sigma ge}^{2}/e+{\sigma \varepsilon }^{2}/re}\times 100\%$$

where ${\sigma g}^{2}$ refers to the genetic variance,$\;{\sigma ge}^{2}$ is the variance of the genotype–environment interactions, ${\sigma \varepsilon }^{2}$ refers to the residuals, e is the number of environments, and r is the number of replications.

To reduce the effects of environmental factors on phenotypes, the BLUP values were calculated using phenotypic data of the RILs in three environments. The BLUP value for every RIL was analyzed using the MLM model implemented in the lme4 package of R (version 3.6.1). The BLUP value was computed according to the formula described by Alvarado et al. [74].

$$Yijlk=u+Linei+Locj+\left(Line\times Loc\right)ij+Rep\left(Loc\right)jl+\varepsilon ijlk$$

‘Yijlk’, ‘u’, ‘Linei’, and ‘Locj’ refer to the KRN phenotype values, intercept, effects, and lth location effects, respectively. ‘(Line × Loc)ij’ is used to denote the interaction between the ith line and the jth location. ‘Rep(Loc)jl’ is the nested effect of the jth copy at the lth location. ‘εijlk’ represents the random effects.

### 5.4. Genotyping-by-Sequencing

DNA was extracted from leaves using the CTAB method by Elshire et al. [75]. The DNA quality was tested using the following methods: (1) The concentration and purity of DNA samples were assessed using NanoDrop (OD260/OD280 ratio); (2) the degree of DNA degradation and potential RNA or protein contamination were analyzed by agarose gel electrophoresis; and (3) fluorescence quantification (Qubit) was used to accurately measure the DNA concentration. DNA samples with an OD260/OD280 ratio ranging from 1.8 to 2.0 and a concentration of 1.0 μg or higher were considered suitable for library construction. Library construction and sequencing were conducted as follows. The restriction endonuclease ApeKI was used for library construction (recognition the sequence GCWGC, where W represents either A or T). The barcode and common junctions were designed according to the ApeKI recognition site to minimize ligation to other genomic DNA fragments [75]. For sequencing, Paired-End150 sequencing was performed using the Illumina HiSeq sequencing platform. After sequencing, the raw reads were filtered to remove adaptor sequences and low-quality reads. [76]. Prior to TASSEL analysis, 80 polyadenylate (polyA) bases were appended to the 3′ end of all sequencing reads, following the method described by McKenna et al. [77]. The maize B73 genome was used as a reference for alignment of the reads using BWA [78]. The alignment results were converted to SAM/BAM files using SAM tools [79]. SNP calls were made using Genome Analysis Toolkit (GATK) software (v4.2) [80]. 

### 5.5. SNP Indentification, Filtration, Annotation, and LD Decay Estimation

SNPs were assessed using Sentieon software (v2021-12-01) [81]. The following criteria were applied to select the SNPs: (1) considering the genotype of each individual at the locus, only genotypes with a GQ value greater than 20 and covered by more than 5 reads were retained; (2) SNPs with multiple alleles were excluded, and only bi-allelic loci were retained; (3) loci with a missing rates higher than 20% were excluded to ensure that for any SNP, at least 80% of the samples in the population were genotyped; (4) loci with a MAF < 0.05 were excluded. Finally, 569,529 high-quality SNPs were obtained. ANNOVAR software [82] was used for the functional annotation of the SNPs. For the degree of LD, the r^2^ between two markers was calculated using the PopLDdecay v3.42 software [83]. The LD decay graph was generated by plotting the paired r^2^ values against the physical distance using the Plot_OnePop.pl software (v2016-04-22) package.

### 5.6. Phylogenetic Tree, PCA, and Population Structure Analysis

Maximum parsimony (MP) was used to compute the distance matrix and construct the phylogenetic tree. PCA was performed using the GCTA 1.5.0 software [84]. Following the completion of the PCA, a two-dimensional representation of the samples included in the analysis was generated using the values of the first two PCs. The population structure analysis was performed using Admixture (version 1.3.0) [85] and the R package was used to visualize the groupings at specific K values or at individual K values.

### 5.7. Genome-Wide Association Analysis

GWAS analyses were conducted using the GEMMA software [86] (www.xzlab.org, accessed on 29 April 2024) with MLM for phenotypes from three environments and BLUP values. A significance threshold of −log10(p) > 4.5 to be used to identify significant SNPs [86]. bedtools v1.7 was used to extract SNPs that meet or exceed the threshold [87]. The MLM was performed using the following formula:

y=Xα+Zβ+Wμ+e

where y represents the phenotypic trait, X represents the incidence matrix for fixed effects, α denotes estimated fixed effect parameter, z is the matrix of SNP, β denotes the effect of SNP, w refers to the random effects, μ represents the predicted random individual, and e stands for a random residual, subject to e~ (0, δe2).

The GWAS results were visualized through Manhattan and Q–Q plots generated using CM plot v3.6.2 [88]. The significance levels of individual SNPs were adjusted using Bonferroni correction [89], a multiple comparison test to control for error rates and false positives.

### 5.8. Linkage Mapping and QTL Analysis

Linkage mapping was conducted using JoinMap 4.0 software [90], with a LOD threshold ≥ 5.0, and the linkage map was drawn using the Perl SVG module. QTL mapping was performed using the composite interval mapping in Windows QTL Cartographer v2.0 [91]. QTLs with an LOD threshold ≥ 2.5 were considered significant. The LOD threshold was determined using a 1000-permutations test at *p* ≤ 0.05 [92]. The proportion of explained PVE attributable to a single QTL was assessed by calculating the square of the R^2^.

### 5.9. Candidate Gene Prediction and Haplotype Analysis

The SNPs identified overlapping the QTL intervals were selected for screening of candidate genes. Functional annotation was conducted using the MaizeGDB, UniProt, and NCBI databases. Candidate gene sequences corresponding to the B73_RefGen_v4 were extracted and compared among the parental lines, Ye107, NA20, YML1218, and CML395. Functional gene motifs were predicted using MEME online software (https://meme-suite.org/meme/, accessed on 5 September 2024). Haplotype analysis of the candidate genes associated with KRN was conducted using Haploview v4.2 software [93]. The haplotypes in which the loci of the significantly associated SNPs are located were identified, and the annotation of the genes within the haplotypes was carried out to identify the functionally related gene loci.

## Figures and Tables

**Table 1:** Statistical analysis of KRN phenotypes.

Populations	Environment	Max	Min	Avg.	SD	CV (%)	Skewness	Kurtosis	H^2^ (%)	Correlation Coefficient (r)
Pop1	23DH	16.00	7.33	11.429	1.939	16.966	−0.149	−0.664	81.63	23DH/223BS = 0.637
(AN20 × Ye107)	23BS	15.56	7.50	11.731	1.823	15.540	−0.363	−0.650		23DH/23YS = 0.633
	23YS	16.33	8.00	11.707	1.862	15.905	−0.216	−0.439		23BS/23YS = 0.643
Pop2	23DH	14.67	8.00	11.339	1.567	13.820	−0.253	−0.692	84.16	23DH/223BS = 0.609
(YML1218 × Ye107)	23BS	15.60	7.50	11.291	1.612	14.277	−0.070	−0.360		23DH/23YS = 0.512
	23YS	14.50	8.00	11.181	1.654	14.793	−0.064	−0.928		23BS/23YS = 0.680
Pop3	23DH	15.40	8.00	11.678	1.768	15.140	−0.071	−0.672	81.40	23DH/223BS = 0.568
(CML395 × Ye107)	23BS	15.00	7.60	11.593	1.587	13.689	−0.072	−0.210		23DH/23YS = 0.604
	23YS	15.33	8.00	11.562	1.426	12.334	−0.169	−0.038		23BS/23YS = 0.614

23DH represents Dehong Prefecture in 2023, 23BS represents Baoshan City in 2023, and 23YS represents Yanshan County in 2023. Max represents maximum, min represents minimum, avg represents average, SD is standard deviation, and CV is coefficient of variation. H^2^ is broad-sense heritability.

**Table 2:** List of QTLs linked to KRN identified in the three RIL subpopulations.

Sub-Populations	QTL	Chr	Environment	Position (cM)	Mapping Interval (bp)	LOD	R^2^ (%)	Additive Effect
pop1	*qKRN7-1*	7	23DH	6.95–11.81	142,993,935–149,543,178	3.4	9.83	0.544
	*qKRN9-1*	9	23DH	0.7–5.72	142,986,935–146,056,154	2.956	9.04	−0.629
	*qKRN7-2*	7	23YS	6.61–10.09	145,964,462–149,665,815	3.335	8.66	0.576
	*qKRN9-2*	9	23YS	0.0.1–5.75	141,014,091–159,769,782	3.488	9.84	−0.587
	*qKRN10-1*	10	23YS	17.54–27.53	41,538,713–42,893,884	3.034	8.78	0.502
	*qKRN3-1*	3	BLUP	19.13–24.28	156,072,764–179,195,272	2.943	7.53	0.321
	*qKRN7-3*	7	BLUP	6.61–11.81	142,993,935–149,665,815	2.831	7.57	0.347
	*qKRN9-3*	9	BLUP	0.01–5.72	142,986,935–159,769,782	3.95	11.39	−0.529
pop2	*qKRN3-2*	3	23YS	12.88–19.34	184,828,793–194,455,629	3.999	12.38	0.519
	*qKRN5-1*	5	23YS	168.13–180.76	58,424,699–65,624,385	3.431	11.29	0.516
	*qKRN6-1*	6	BLUP	215.17–215.54	12,557,157–21,457,547	2.803	7.92	0.428
	*qKRN8-1*	8	BLUP	15.87–24.24	117,852,363–151,803,566	3.202	9.69	0.382
pop3	*qKRN2-1*	2	23DH	35.39–25.88	165,579,158–168,473,058	3.155	6.2	−1.075
	*qKRN5-2*	5	23DH	282.32–286.35	203,965,201–209,017,558	2.638	6.39	−0.509
	*qKRN6-2*	6	23BS	0.1–2.26	45,543,817–46,520,130	4.155	7.27	0.495
	*qKRN9-4*	9	23BS	122.31–125.3	21,498,005–22,427,085	3.156	5.45	−0.438
	*qKRN3-3*	3	23YS	518.65–549.88	155,838,862–163,336,986	4.075	10.87	−0.687
	*qKRN5-3*	5	23YS	18.15–58.35	141,484,114–150,066,634	5.203	14.47	−0.797
	*qKRN8-2*	8	23YS	26.11–40.4	33,141,712–37,541,631	6.367	15.96	0.86
	*qKRN8-3*	8	23YS	57.24–65.65	99,997,661–101,133,204	3.522	7.32	−0.579
	*qKRN1-1*	1	BLUP	493.05–511.74	165,995,163–170,331,469	3.42	7.9	−0.384
	*qKRN7-4*	7	BLUP	387.86–391.31	5,817,348–80,788,780	2.567	6.72	0.474

**Table 3:** Candidate genes identified through co-localization analysis by GWAS and QTL mapping.

SNP/QTL	Chr	Position	Mapping Interval of QTL (bp)	Candidate Gene	Candidate Gene Range (bp)	Functional Annotation
178,487,003	3	178,487,003 bp	178,466,983–178,507,003	*Zm00001d042733*	178,487,316–178,488,789	UCH
*qKRN3-1*		22.86 cM	156,072,764–179,195,272			
178,487,003	3	178,487,003 bp	178,466,983–178,507,003	*Zm00001d042735*	178,493,943–178,494,617	ATL
*qKRN3-1*		22.86 cM	156,072,764–179,195,272			

**Table 4:** Important haplotypes of candidate genes associated with KRN.

Gene ID	Position	Haplotype	Hap_Sample_Num ^1^
*Zm00001d042733*	SNP3-178487003	Hap1 (CGTT)	46
		Hap2 (CGTG)	21
		Hap3 (CACG)	47
*Zm00001d042735*	SNP3-178487003	Hap1 (CTCG)	135
		Hap2 (CCCG)	44
		Hap3 (TTGC)	96

Note: hap_sample_num ^1^ refers to the number of identical haplotypes.

**Table 5:** Details of parental materials for breeding MPP.

Parental Lines	Pedigree	Heterotic Groups	Ecotype
YE107	Derived from US hybrid DeKalb XL80	Reid	Temperate
AN20	Derived from US hybrid	nonReid	Subtropical
YML1218	HuangZhaoShi×WeiChun	nonReid	Temperate
CML395	90323B-1-B-1-B*4-1-1-2-1(DH)	nonReid	Tropical

**Table 6:** Information about the experimental locations.

Location	Latitude and Longitude ^1^	Crop Season	Soil Types ^1^	Month	Average Rain Fall (mm) ^2^
23DH	98°60′ N, 24°4′ E	summer	red	May–October	130.65
23BS	99°20′ N, 25°1′ E	summer	red	May–October	25.27
23YS	23°60′ N, 104°4′ E	summer	red	May–October	125.72

^1^: Dates from China Soil Database (vdb3.soil.csdb.cn, accessed on 17 November 2024), ^2^: Dates from National Historical Temperatures (www.tianqi24.com, accessed on 17 November 2024).

## Data Availability

The data presented in this study are available on request from the corresponding author.

## Supplementary Materials

The following supporting information can be downloaded at https://www.mdpi.com/article/10.3390/plants13233308/s1, Table S1: Details of SNPs significantly associated with KRN identified during GWAS; Table S2: Candidate Gene Information Table; Table S3: Summary of Genetic Linkage Groups of three RIL populations; Figure S1: QTL Mapping; Figure S2: genetic linkage map.
