# Supplementary figures and images for: Combined Genome-Wide Association Study and Linkage Analysis for Mining Candidate Genes for the Kernel Row Number in Maize (Zea mays L.)
Source: Plants (Basel). 2024 Nov 26;13(23):3308. doi: 10.3390/plants13233308 (PMC11644245; doi:10.3390/plants13233308)

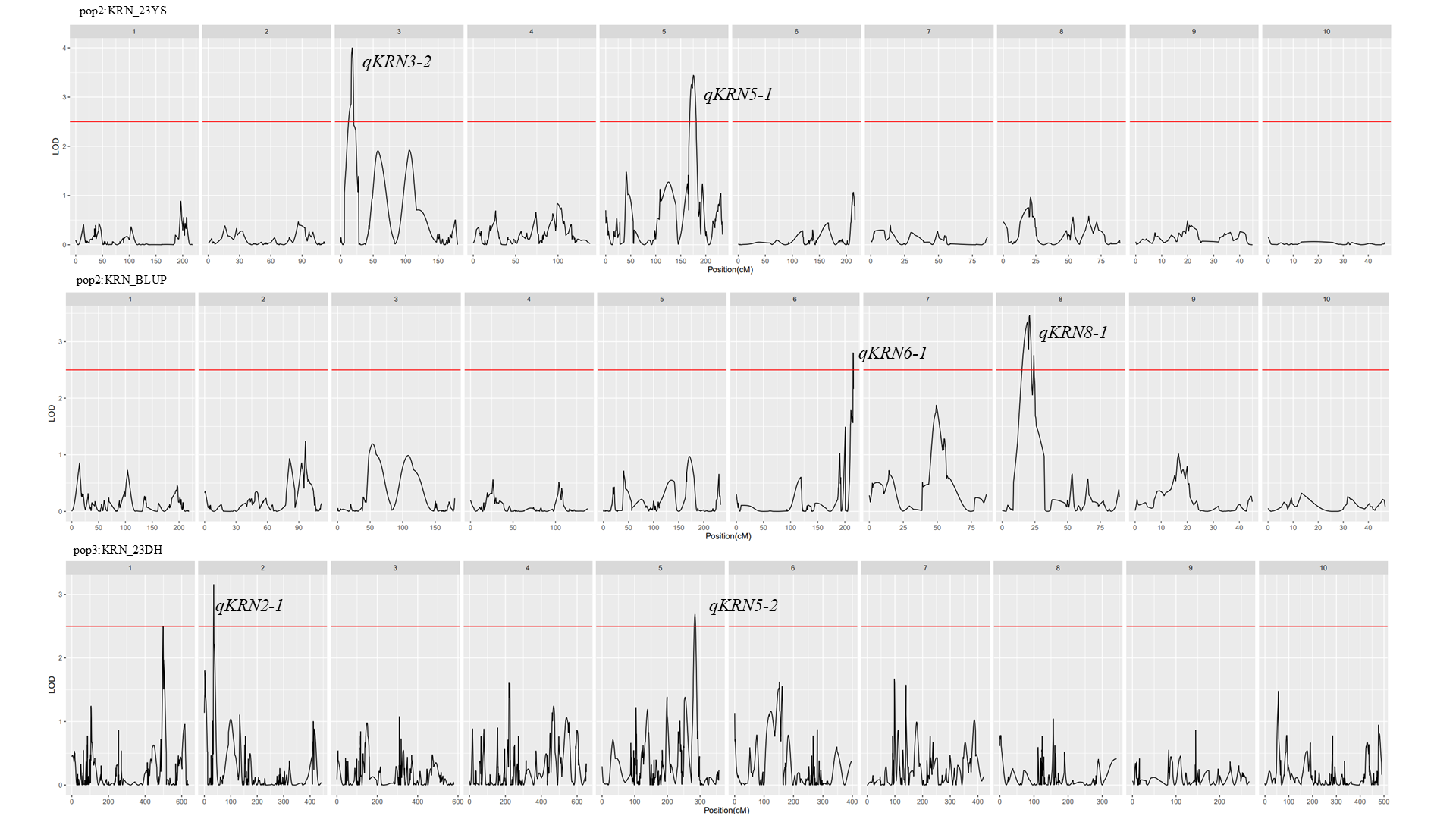

Supplement: Supplementary file 1 [file plants-13-03308-s001.zip › plants-3321559-supplementary/FigureS1/QTL Mapping (2).png]

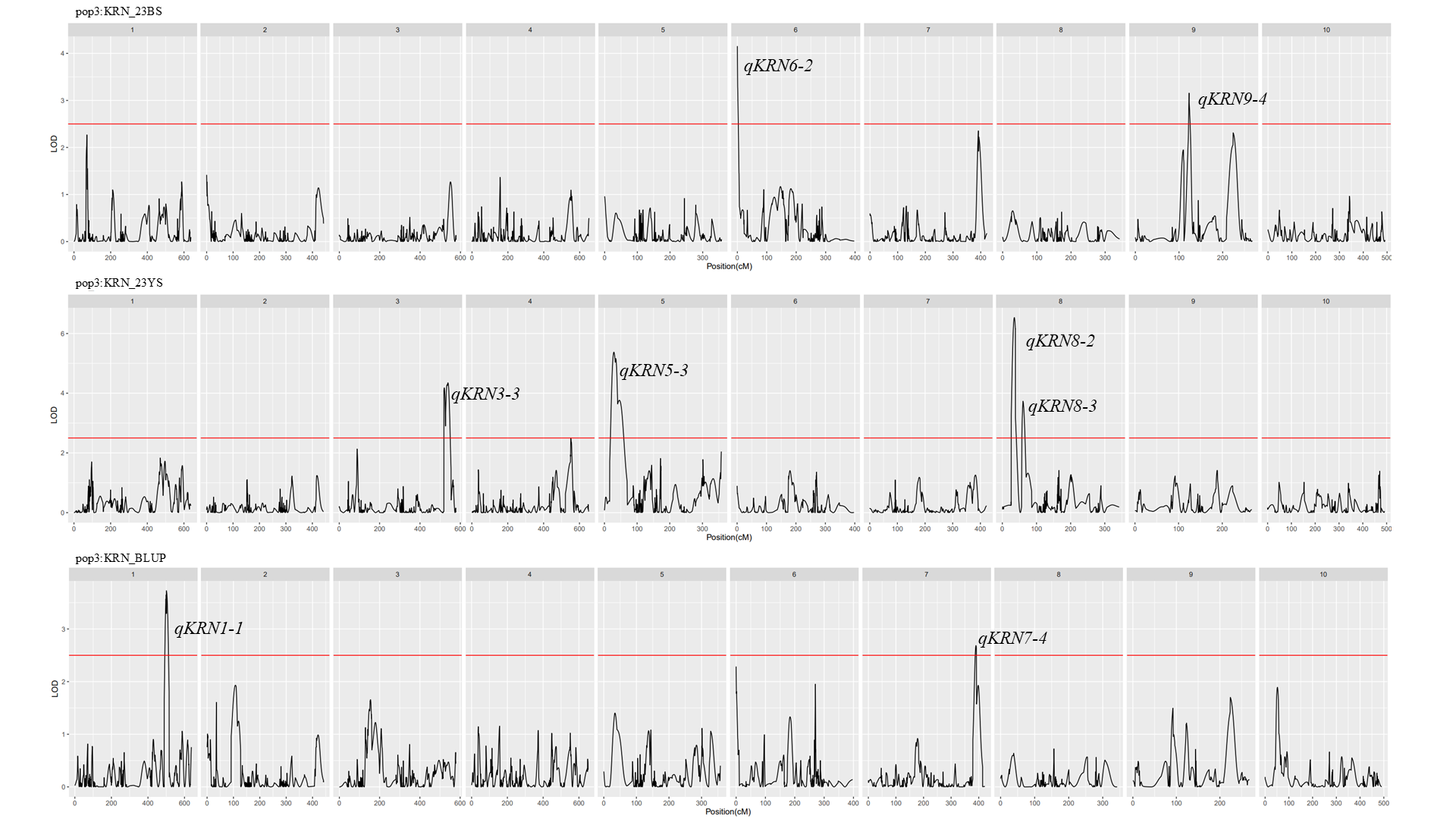

Supplement: Supplementary file 1 [file plants-13-03308-s001.zip › plants-3321559-supplementary/FigureS1/QTL Mapping (3).png]

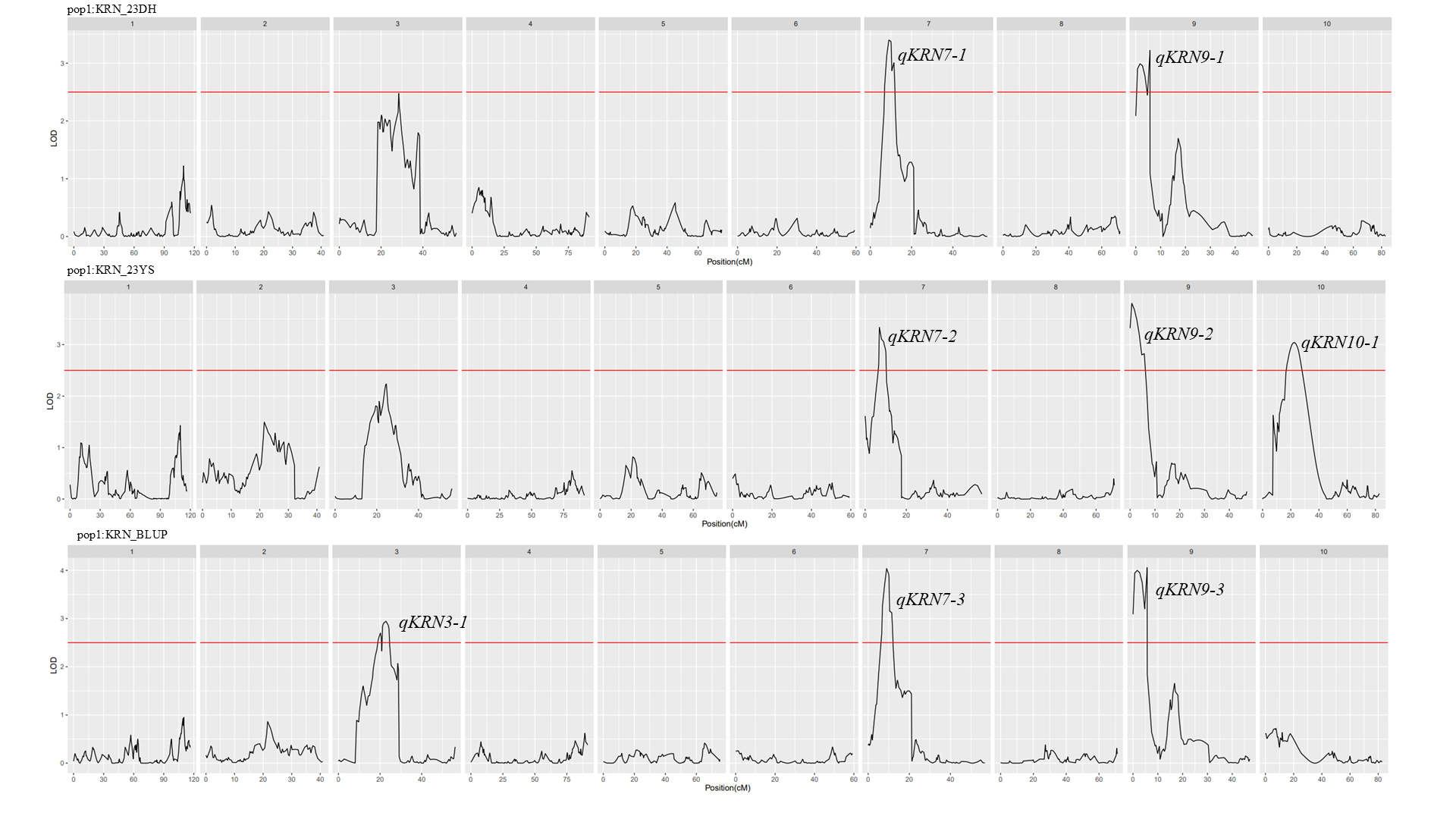

Supplement: Supplementary file 1 [file plants-13-03308-s001.zip › plants-3321559-supplementary/FigureS1/QTL Mapping.png]

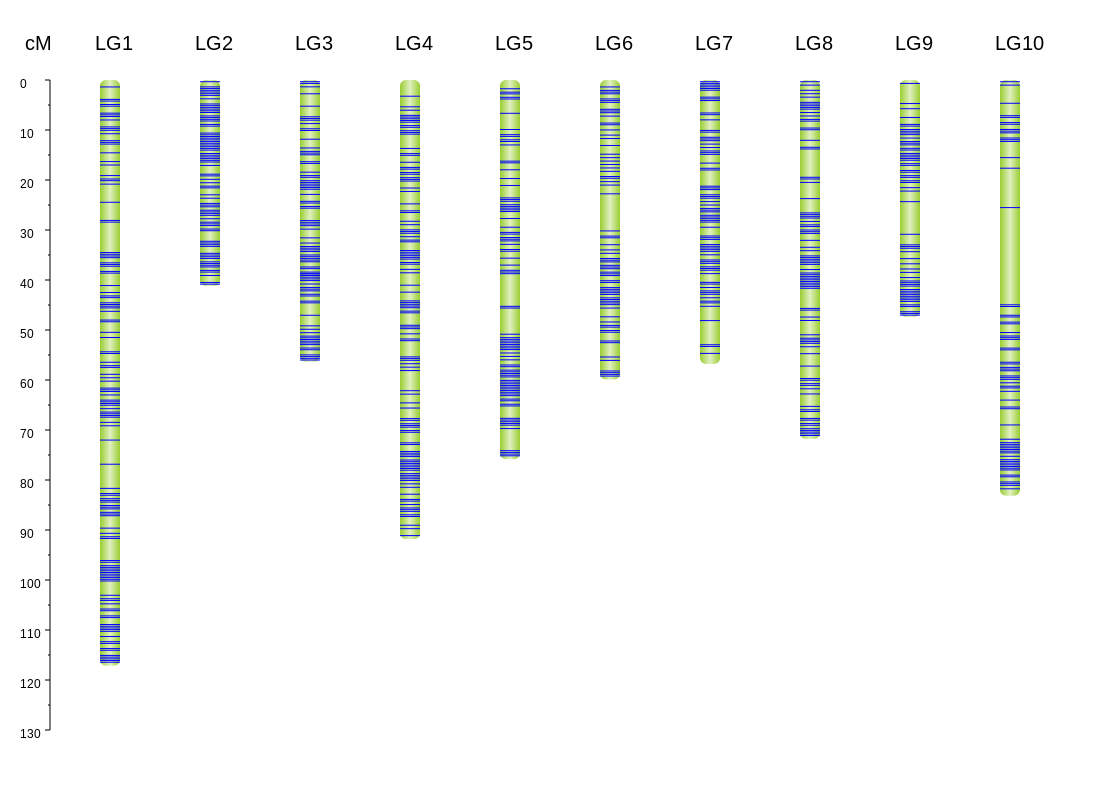

Supplement: Supplementary file 1 [file plants-13-03308-s001.zip › plants-3321559-supplementary/FigureS2/AN20.png]

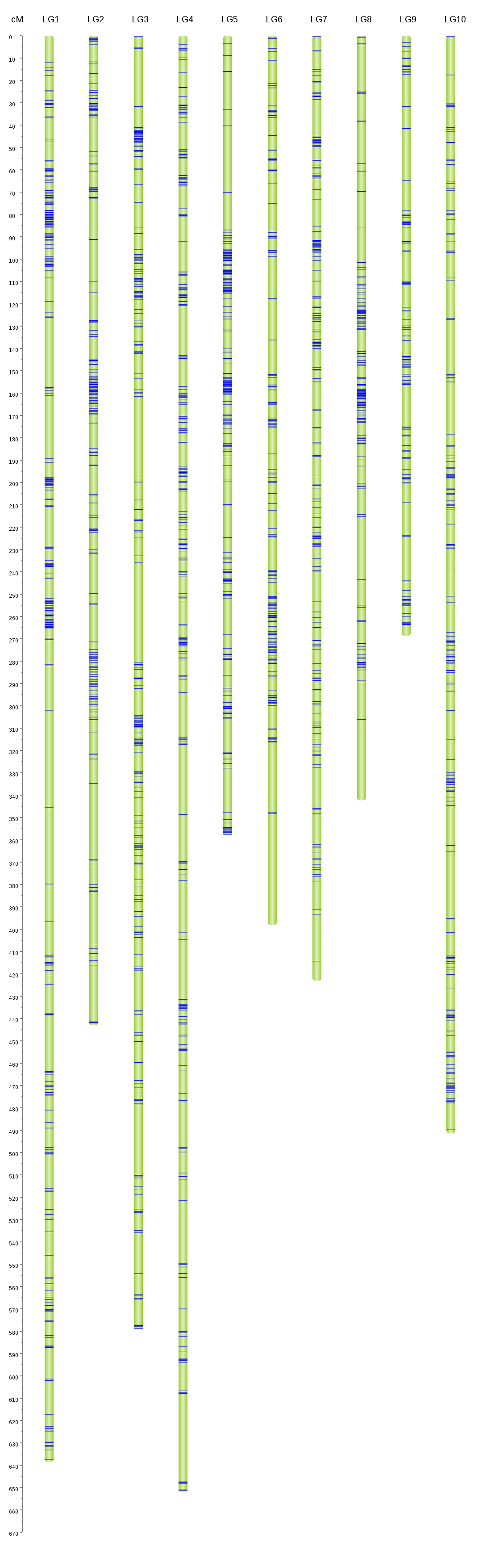

Supplement: Supplementary file 1 [file plants-13-03308-s001.zip › plants-3321559-supplementary/FigureS2/CML395.png]

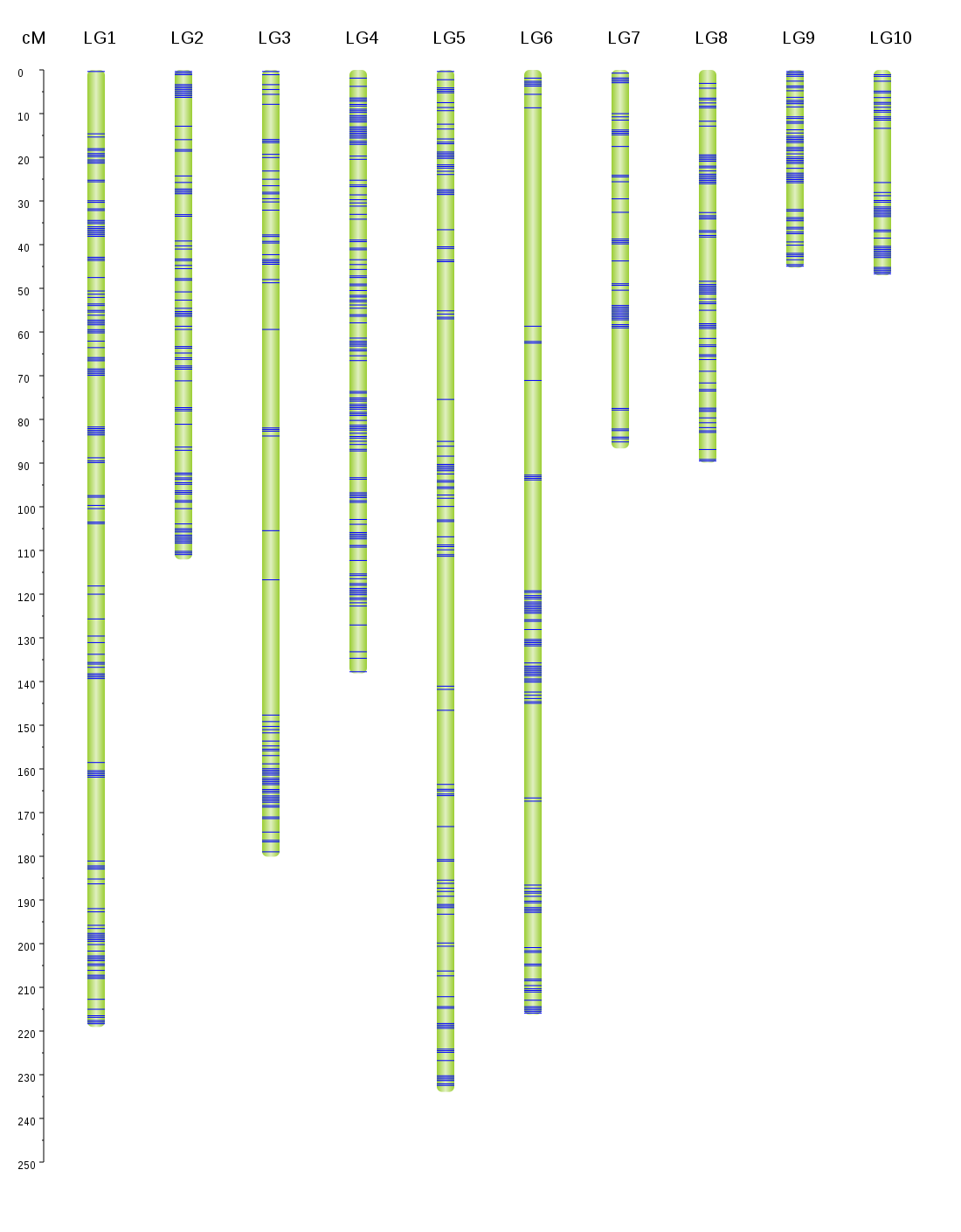

Supplement: Supplementary file 1 [file plants-13-03308-s001.zip › plants-3321559-supplementary/FigureS2/YML1218.png]
